# The Use of Thoracic Ultrasound to Predict Transudative and Exudative Pleural Effusion

**DOI:** 10.24908/pocus.v6i2.15193

**Published:** 2021-11-23

**Authors:** Peter T Evans, Robert S. Zhang, Yulei Cao, Sean Breslin, Nova Panebianco, Cameron M. Baston, David M Dibardino

**Affiliations:** 1 Department of Medicine, University of Pennsylvania Philadelphia, PA; 2 Drexel University College of Medicine Philadelphia, PA; 3 Department of Emergency Medicine, University of Pennsylvania Philadelphia, PA; 4 Section of Interventional Pulmonology and Thoracic Oncology, Division of Pulmonary, Allergy and Critical Care, University of Pennsylvania Philadelphia, PA

**Keywords:** Pleural Effusion, Ultrasound, Thoracentesis

## Abstract

**Objectives: **Pleural effusion is a common reason for hospital admission with thoracentesis often required to diagnose an underlying cause. This study aimed to determine if the imaging characteristics of TUS effectively differentiates between transudative and exudative pleural fluid. **Methods: **Patients undergoing TUS with pleural fluid analysis were retrospectively identified at a single center between July 2016 and March 2018. TUS images were interpreted and characterized by established criteria. We determined diagnostic performance characteristics of image criteria to distinguish transudative from exudative pleural effusions. **Results: **166 patients underwent thoracentesis for fluid analysis of which 48% had a known malignancy. 74% of the pleural effusions were characterized as exudative by Light’s Criteria. TUS demonstrated anechoic effusions in 118 (71%) of samples. The presences of septations on TUS was highly specific in for exudative effusions (95.2%) with high positive predictive values (89.5%) and likelihood ratio (2.85). No TUS characteristics, even when adjusting for patient characteristics such as heart failure or malignancy, were sensitive for exudative effusions. **Conclusions: **Among our cohort, anechoic images did not allow reliable differentiation between transudative and exudative fluid. Presence of complex septated or complex homogenous appearance was high specific and predictive of exudative fluid.

## Background

Over 1.5 million people develop pleural effusion each year and there is an estimated prevalence of 60% in the intensive care unit (ICU) 1, 2. Thoracic ultrasound (TUS) has been shown to be more sensitive than chest radiography and physical examination for pleural effusion and is routinely used to detect and evaluate pleural effusions 3. Although the use of TUS to guide thoracentesis has improved procedural safety and should be considered standard of care, there are still risks associated with the procedure 4, 5. The ability to predict the chemical characteristics of a pleural effusion prior to sampling may impact subsequent management and potentially decrease the need for thoracentesis and associated procedural risks. There have been limited data that have examined the diagnostic accuracy of TUS’s ability to differentiate a transudative from an exudative effusion. Most prior studies have shown that TUS was reliable in identifying exudative effusions but not transudative effusion; however, outside of a recent evaluation by Shkolnik and Asciak et al, these studies were older with different technology, and often had smaller sample sizes 6, 7, 8, 9. 

In this study, we examined the diagnostic performance of TUS in predicting transudative and exudative effusions. 

## Methods

### Study Population and Data Collection

This study was approved by the University of Pennsylvania Institutional Review Board (IRB number 828853). The requirement for written informed consent was waived by the board. We retrospectively identified consecutive patients who were evaluated by the procedure service for consideration for thoracentesis with archived pleural effusion images at a single large academic center between July 2016 and March 2018. At our center thoracenteses performed on the acute care medicine floors are performed by a dedicated procedure service staffed by Nurse Practitioners and Physician Assistants supervised by Pulmonary and Critical Care faculty. The service performs approximately 600 thoracenteses per year.

Patient clinical, demographic and pleural fluid analysis data were abstracted via chart review by physicians. TUS was performed with a Sonosite Xporte machine using a phased-array sector transducer in the abdominal preset. The other acquisition specifics, including gain, depth, and patient positioning were left to the discretion of the operator. TUS was performed by multiple operators with experience in lung ultrasound. All pleural effusions included a short video clip and/or still images identifying the diaphragm, pleural effusion and lung parenchyma. The TUS images were analyzed retrospectively by board certified pulmonary and critical care physicians (CB and DD) who were blinded to the clinical data (including Light’s criteria) and prior image interpretation. Disagreement between two primary reviewers was arbitrated by an ultrasound fellowship trained physician (NP). 

**Figure 1:**
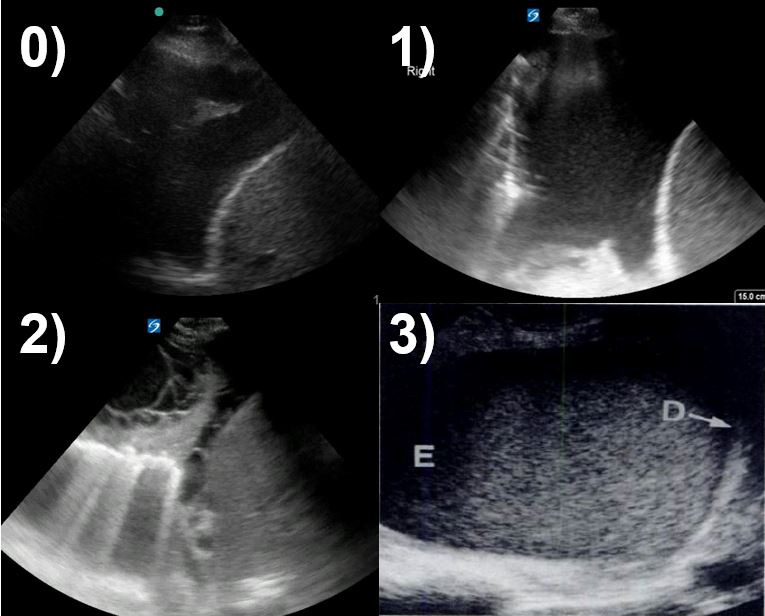
Sample pleural fluid images. 0, anechoic – no echoes present between the pleura and diaphragm; 1, complex, non-septated – increased echogenicity of the space between the pleura and diaphragm, without clear hyperechoic linear findings to suggest septation; 2, complex, septated – echogenic linear structures present in the space between the pleura and diaphragm; 3, homogenously echogenic – echogenic material filling the entire space between the pleura and diaphragm.

The TUS images of the pleural effusions were classified by previously published criteria 6, 7. Anechoic was defined by no echoes present between the visceral pleural and diaphragm. Complex, nonseptated was defined by an increased echogenicity of the space between the visceral pleura and diaphragm, without clear hyperechoic linear findings to suggest septation. Complex, septated was defined as echogenic linear structures presenting in the space between the visceral pleura and diaphragm. Homogenously echogenic was defined as echogenic material filling the entire space between pleural and diaphragm. Figure 1. demonstrates representative imaging.

The decision to complete a thoracentesis was made by the primary team (rather than procedure service performing the imaging and thoracentesis), unless TUS demonstrated lack of adequate volume for safe drainage or other safety concerns. Pleural fluid was sent for analysis and specific tests were ordered by the primary team and typically included: gram stain and culture, pH, lactate dehydrogenase (LDH), cell count and differential, total protein, glucose, and cytology. It was standard practice for the primary team to send serum LDH and total protein at the time of the procedure. 

Pleural effusions were classified as exudative or transudative based on the Light’s criteria 10. An exudative effusion was defined as a parapneumonic effusion if there was evidence of an adjacent pneumonia or lung abscess, positive gram stain or culture, or biochemical evidence of inflammation (pH <7.20 or glucose <60). Empyema was identified if the effusion had frank purulent drainage. An exudative effusion was defined as malignant if there was evidence based on cytology, flow cytometry, histology or known cancer without an alternative cause of the effusion. 

### Statistical Analysis

Continuous variables are presented as mean ± standard deviation (SD) or median with interquartile range (IQR) for skewed data. Categorical data are expressed as frequencies and proportions. Logistic regression was used to determine the relationship between sonographic findings and final categorization of the effusion based on the Light’s criteria. Cohen’s kappa coefficient was calculated to determine the inter-observer agreement for the sonographic score. Two by two tables were created to determine the sensitivity, specificity, positive predictive value and negative predictive value. Analyses were performed using Stata version 15 (College Station, TX: StataCorp LLC). 

## Results

**Figure 2:**
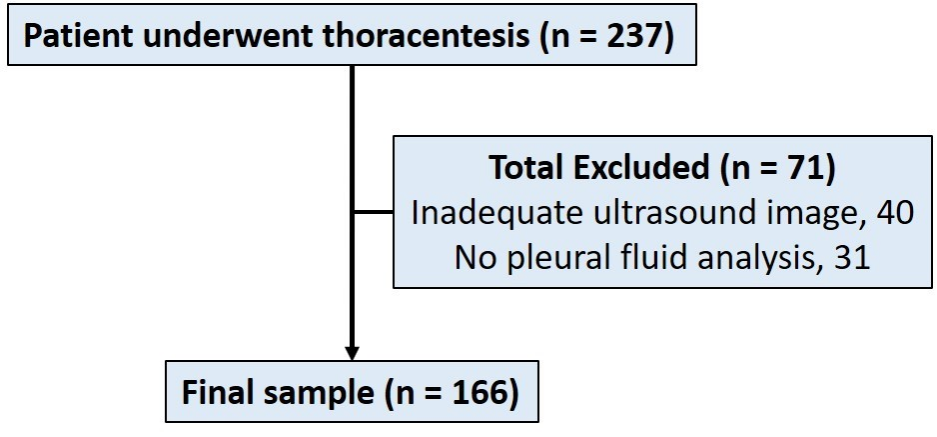
Patient flow chart.

Our analysis included 166 thoracenteses performed (Figure 2). Patients’ mean BMI was 29.4 and 41.5% of patients were female. Patient demographics characteristics are described in Table 1.

**Table 1:** Baseline characteristics

	**N=166**
BMI, kg/m (mean, SD)	29 (18)
Female sex (%)	69 (41.5%)
Malignancy	81 (48%)
Infection	9 (5%)
Hepatohydrothorax	22 (14%)
Heart failure	11 (7%)

**Figure 3:**
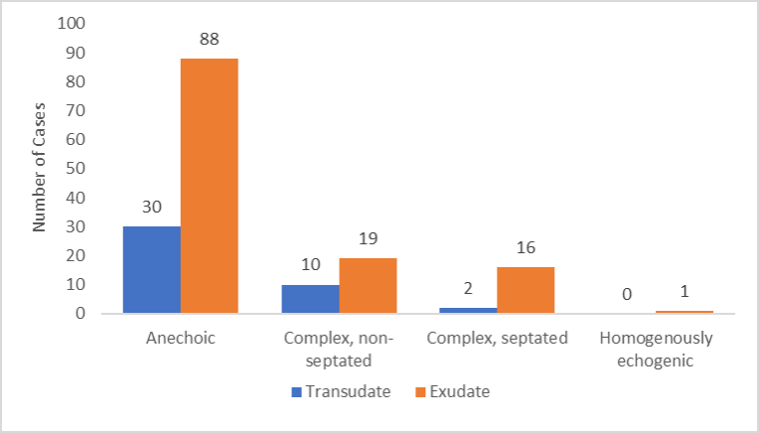
Distribution of effusion studies by thoracic ultrasound image characteristics.

Ultrasound images included 118 images that were anechoic, 29 complex and non-septated, 18 complex and septated, and 1 homogenously echogenic. Figure 3 displays the relationship between the TUS findings with a pleural effusion classification of transudative vs exudative. After laboratory analysis, 124 (74%) were exudative by Light’s criteria, while 42 (25%) were transudative. The etiologies of effusion included malignancy (48%), infection (5%), hepatohydrothorax (14%), heart failure (7%) and other (26%). The breakdown of ultrasound image characteristic and fluid sampling is outline in Table 2.

**Table 2:** Distribution of Pleural Diagnoses by Thoracic Ultrasound.

	Transudate	Exudate	Total
Anechoic	30	88	118
Complex, non-septated	10	19	29
Complex, septated	2	16	18
Homogenously echogenic	0	1	1
Total	42	124	166

Sensitivity of a nonzero sonographic score for an exudate was 29% (95% CI 21-38%), while specificity was 71.4% (95% CI 55-84%). The positive likelihood ratio (LR) of an exudate being present on a thoracentesis following a nonzero sonographic score was 1.0 (95% CI 0.6-1.7). The presence of septations had a specificity of 95.2% (95% CI 84-99%) for an exudate. The positive LR of an exudate being present on thoracentesis following TUS findings of septations was 2.85 (0.7-12). After excluding patients for whom only still images were available, the specificity, PPV and positive likelihood ratio of the presence of septations increased to 96% (95% CI 80-99), 94% (95% CI 73-99), and 4.7 (95% CI 0.66-33.8) respectively. The diagnostic performance of TUS to predict transudative and exudative effusions are described in Table 3 and Table 4. Patient conditions including as decompensated heart failure, hepatohydrothorax, and malignancy had wide distributions of pleural diagnosis (Tables 5-7).

**Table 3:** Diagnostic Performance of Ultrasound Score to Predict Exudative Effusions.

	Sensitivity (95% CI)	Specificity (95% CI)	PPV (95% CI)	NPV (95% CI)	Positive LR (95% CI)
Nonzero sonographic score (%)	29 (21-38)	71 (55-84)	74 (60-86)	25 (18-34)	1.0 (0.6-1.7)
Presence of Septations (%)	13(8-21)	95 (84-99)	89 (67-98)	27 (20-35)	2.9 (0.7-12)
Anechoic Effusion Resulting in Transudate	71 (55-84)	29 (21-38)	25 (18-34)	75 (60-86)	1.0 (0.8-1.2)

**Table 4:** Diagnostic Performance of Ultrasound Score to Predict Exudative Effusions (Video Only)

	Sensitivity (95% CI)	Specificity (95% CI)	PPV (95% CI)	NPV (95% CI)	Positive LR (95% CI)
Nonzero sonographic score (%)	37 (27-48)	68 (46-85)	80 (65-91)	23 (14-36)	1.1 (0.6-2.2)
Presence of Septations (%)	19 (11-28)	96 (80-99)	94 (73-99)	24 (16-34)	4.7 (0.66-33.8)
Anechoic Effusion Resulting in Transudate	68 (46-85)	37 (27-85)	23 (14-34)	80 (56-91)	1.1 (0.8-1.5)

**Table 5:** Distribution of Pleural Diagnoses by Thoracic Ultrasound in Patients with Decompensated Heart Failure

	Transudate	Exudate	Total
Anechoic	4	4	8
Complex, non-septated	1	2	3
Complex, septated	0	0	0
Homogenously echogenic	0	0	0
Total	8	3	11

**Table 6:** Distribution of Pleural Diagnoses by Thoracic Ultrasound in Patients with Hepatohydrothorax

	Transudate	Exudate	Total
Anechoic	6	7	13
Complex, non-septated	5	1	6
Complex, septated	1	2	3
Homogenously echogenic	0	0	0
Total	12	10	22

**Table 7:** Distribution of Pleural Diagnoses by Thoracic Ultrasound in Patients with Malignancy

	Transudate	Exudate	Total
Anechoic	7	50	57
Complex, non-septated	0	14	14
Complex, septated	1	8	9
Homogenously echogenic	0	1	1
Total	8	73	81

There was disagreement between the two primary raters in 18 (11%) cases, most commonly due to assessment of the presence of gain artifacts. Overall agreement between the two raters was substantial with a Cohen’s kappa of 0.75. Overall agreement between the two raters between TUS videos only was almost perfect with a Cohen’s kappa of 0.81. Interrater reliability was decreased between the two raters when only ultrasound still images were used with a Cohen’s kappa of 0.67.

## Discussion

In a cohort of 166 pleural effusions, the presence of septations on TUS evaluation was highly specific for exudative fluid, though not predictive for a specific etiology. Nonseptated echoes in the pleural space was only moderately specific, and not sensitive for exudative fluid. Finding anechoic fluid did not reliably differentiate transudative or exudative fluid. 

Previous studies have examined the relationship between ultrasound images and pleural fluid characteristics. Yang et al. evaluated a cohort of 320 patients with pleural effusions, finding high sensitivity, but poor specificity of anechoic effusions for transudative effusions 6. In our study, we found anechoic effusions to be only moderately sensitive for transudative effusions. While 23 (21%) of patients had heart failure or hepatohydrothorax as the etiology of their pleural effusion, half of these patients had exudative effusions. Although the chemical analysis of these pleural effusions were exudative, these effusions may have been transudates that became concentrated into exudates through diuresis and may explain the low sensitivity of anechoic effusions for transudates in our population. Unlike findings in subsequent studies, patients with any pleural complexity had exclusively exudative effusions. Svigals et al. described 66 patients with parapneumonic effusions, finding TUS had sensitivity of 69.2% (95% CI 48.2% to 85.7%) and specificity of 90.0% (95% CI 76.3% to 97.2%) 8. In a cohort of 126 patients with transudative pleural effusions, Chen et al. found that an anechoic pattern was present in 45% (57/127), while a complex nonseptated pattern in 55% (70/127); transudative fluid was never complex septated or homogenously echogenic 11. Asciak et al. found that of 85 patients with echogenic effusions, six (7.1%) had transudates, leaving the specificity of echogenicity to identify exudates from transudates as 57.1% 9. A recent evaluation of 300 pleural effusions in 285 patients found that detection of septations or homogenous complexity was 94% specific and carried a 96% positive predictive value for exudative fluid. Additionally, anechoic fluid did not reliably predict the presence of transudative fluid ^7^. Outside of a much lower sensitivity (21% vs 70%) for complex effusions our findings support the conclusions of that study, in a different patient population at a different center. 

These findings support and extends existing primary literature in several ways. The performance characteristics generally mirrored that of the most recent evaluation of TUS by Shkolnik et al, supporting the high specific and PPV of septations for exudative fluid, though the confidence interval for the positive LR cross 1.0 and are wide 7. Additionally, the present study was enriched in patients with known malignancy. Performance characteristics of TUS did not differ from a broader cohort in this subgroup, suggesting diagnostic thoracentesis would likely be required for definitive diagnosis of a new pleural effusion in a patient with known malignancy. 

The study also highlights the importance of video images for interpretation with increases of both testing characteristics and inter-rater agreement when compared to evaluation with still images. When disagreement occurred between the two primary raters, it was most frequently in still images and often due to assessment of the presence of gain artifacts.

Future studies should focus further standardization of machine settings and scanning protocols. Additionally, our study highlights the need to identify other ultrasound imaging characteristics to improve diagnostic performance. Studies have identified the presence of pleural masses, pleural thickening >1 cm, pleural nodularity and diaphragmatic thickening >7 mm as TUS findings that are highly suggestive of malignant pleural effusion disease 12, 13. Incorporation of other imaging features with the standard pleural fluid image characterization may increase diagnostic performance.

Limitations of our study include describing only a single center, which may limit generalizability, though had similar findings to other studies at other centers. Certain pleural fluid diagnoses may have been more common at our institution and less common at other locations. Some patients in our series did not have a complete set of diagnostic tests reviewed for the study because the tests were ordered by the physician responsible for the patient’s care at the time of thoracentesis. This may have led to some selection bias (for example pleural effusions not requiring fluid analysis because it was thought to be due to heart failure). Lastly, while there was high degrees of inter-rater reliability, it was not perfect. This highlights the potential need for strict criteria for classification of effusion, and potentially the limitation of retrospective video review in the absence of a standardized scanning methodology. Though used by experienced operators that frequently work together, machine settings including gain were not always consistent. Insufficient gain could inappropriately characterize a complex, nonseptated effusion as anechoic while with too much gain faint artifacts will make anechoic fluid appear to have internal echoes 14. An expert may be able to differentiate the patterns of artifact overgain from actual echogenicity based on the movement of echoes within the fluid, but this has not been tested in published literature. Ideally, gain would be standardized using a known echogenic quantity such as the blood inside a vessel, but those are not always visible in the same window as a pleural effusion. Similarly, image acquisition is operator dependent. While we evaluated rater inter-rater agreement, examining differences between ultrasound acquisition by user may have added value.

## Conclusion

TUS is already part of the standard of care in thoracentesis and using that information beyond simply marking the puncture site can add value. Our analysis suggests that anechoic pleural fluid images do not reliably distinguish between transudative and exudative fluid. While features such as complex septation or homogenous echogenicity are high supportive of exudative fluid, further research is needed to identify other features that would increase diagnostic accuracy. 

## Disclosures

Dr. Cameron Baston receives royalty payments from McGraw Hill for a textbook on POCUS.
